# Liposarcoma of the forearm in a man with type 1 neurofibromatosis: a case report

**DOI:** 10.1186/1752-1947-3-7071

**Published:** 2009-04-29

**Authors:** Markus Dietmar Schofer, Mohammed Yousef Abu-Safieh, Jürgen Paletta, Susanne Fuchs-Winkelmann, Bilal Farouk El-Zayat

**Affiliations:** 1 Department of Orthopaedics, University Hospital Marburg, Baldingerstrasse, 35033 Marburg, Germany

## Abstract

**Introduction:**

The combination of neurofibromatosis and liposarcoma is very rare. We present a case of a dedifferentiated liposarcoma in the forearm, as a complication in a patient with neurofibromatosis type 1.

**Case presentation:**

A Caucasian man with neurofibromatosis type 1 presented at our clinic complaining of a slow growing swelling on his left forearm over a period of one and a half years. Clinical examination and history pointed to malignancy. Radiological examination inclusive of magnetic resonance imaging and positron emission tomography confirmed our suspicion. A final diagnosis of dedifferentiated high-grade liposarcoma with axillary lymph node metastases was established after a pathological examination of a tumour biopsy. The consulting tumour board recommended either an elbow exarticulation or an accurate radical local resection including the metastatic axillary lymph nodes. Fortunately, we were able to perform an R-zero resection and the forearm could be saved. The treatment was completed with postoperative radiotherapy of the left forearm's operative bed, the left axillary and the supraclavicular regions. The patient decided against adjuvant chemotherapy.

**Conclusion:**

Liposarcoma complicating neurofibromatosis type 1 is a very rare combination. Up to now, only five cases have been reported in the literature. We are adding a new case to this short list to stress the importance of early recognition. It is the first known case with this disease combination in an upper extremity. Liposarcoma is usually treated by surgery followed by radiotherapy. The role of chemotherapy is controversial and should be based on a decision made on a case-by-case basis.

## Introduction

Soft-tissue sarcomas in adults associated with a clinically identified genetic disease represent 2.8% of all cases [1]. Neurofibromatosis type 1 (NF1) is a disorder of autosomal dominant inheritance due to an abnormal gene of chromosome 17 (q11.2). It is characterized by multiple cutaneous neurofibromas, soft papillomas, café-au-lait macules, freckling in the axillary or inguinal areas, optic glioma, iris hamartomas, sphenoid dysplasia and first-degree relative as a risk factor [2]. Cancer is the main cause of early death in such groups [1]-[3]. However, liposarcoma complicating NF1 is a rare entity. Up to now, few cases have been reported in the literature [4]-[8]. We present a case of forearm liposarcoma in a patient with NF1.

## Case presentation

A 41-year-old Caucasian man, known to have generalized NF1 since the age of 21, presented at our clinic complaining of a swelling in his left forearm. He reported that his complaint started 1.5 years previously, when he noticed a slow-growing swelling. The patient was assured that the swelling was a simple lipoma that could be electively resectioned. The patient's history revealed no preceding trauma or inflammation. The swelling was not painful. His family history revealed no similar conditions, but his father and grandmother were operated on for bowel carcinoma. According to the physical examination, the patient had generalized cutaneous neurofibromas all over his body and some papillomas in his mouth. He is of average build and was of normal health and without any other systemic diseases. Local examination showed a hard, lemon-sized, non-movable tumour in the ventral aspect of the left middle forearm. The skin was not affected. The range of movement of the left forearm and hand were free, as was the range of movement at the wrist.

**Figure 1:**
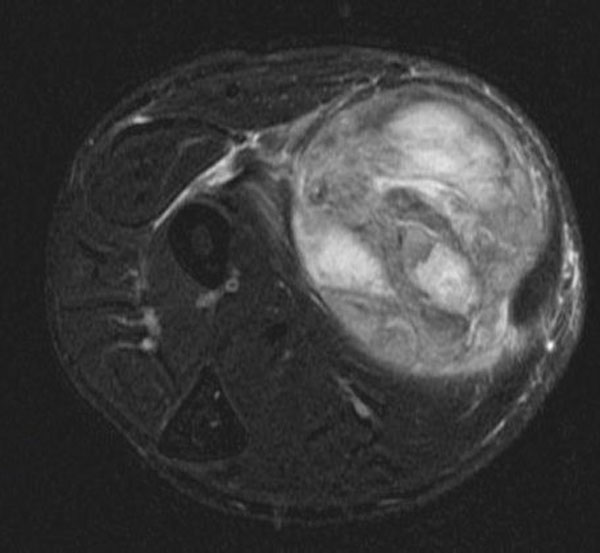
**Axial T2-weighted magnetic resonance image with fat saturation of the left forearm showed a well defined, highly inhomogeneous tumour with liquid- and fat-equivalent parts displacing the surrounding tissue**.

Radiological examination started with X-ray imaging of the left forearm. It showed a soft tissue tumour with no bone affection. Ultrasonography showed a 91 × 52 × 50 mm inhomogeneous condensed tumour with a well-defined border. Contrast-enhanced magnetic resonance imaging (MRI) showed a 95 × 55 × 49 mm well-defined, highly inhomogeneous tumour with liquid and fat equivalent parts. It displaced surrounding tissue (Figure 1).

Following radiological criteria, the tumour was suspected to be malignant. Therefore, we took an open biopsy of the central tumour region. Pathological examination revealed a heterogeneous tumour. There was a region of myxoid ground matrix with parallel elongated spindle-shaped cells with hyperchromatic nuclei and another region of enlarged cells with clear cytoplasm and hyperchromatic and deformed nuclei. We identified multiple apoptosis and some mitosis. Some regions of the tumour were necrotic. Moreover, thrombosed capillary vessels and necrosis were observed. In better differentiated tumour regions, there were lipoblasts with typical polylobulated hyperchromatic nuclei and circumscribed nucleolus vacuoles. According to immunohistochemistry, the lipoblast cells in the well-differentiated component tested positive for S100 protein, but the solid regions were negative. The S100 protein subtype is known as an immunohistopathological marker for defining tumour origins. In conclusion, a diagnosis of a malignant dedifferentiated high-grade liposarcoma was established.

**Figure 2:**
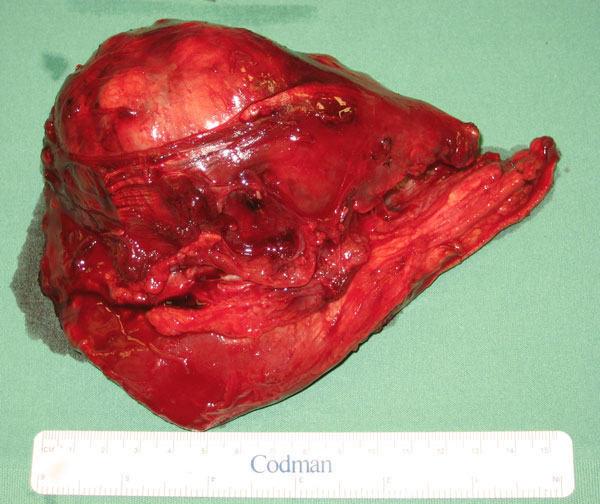
**The tumour after resection**.

The screening for metastases included computed tomography of the chest and abdomen, as well as bone scintigraphy and a fluorine-18-deoxyglucose-positron emission tomography examination. We found a lymph node metastasis in the left axilla, which was verified by an ultrasound examination. It was irregularly enlarged with a diameter of 23 mm. After consulting the tumour board in our hospital, two surgical options were discussed. The first was to perform a local radical tumour resection with a safety margin, on condition that there was no local spreading. This option was supported by the patient's wish to save his forearm. The second option was to perform an elbow exarticulation. Further treatment should have included radiotherapy and chemotherapy. Intraoperatively, a tumour-free edge was revealed, which allowed the surgeon to proceed towards the tumour resection, with a safety margin (Figure 2). Because the tumour was infiltrating the flexor carpi radialis muscle, both were totally resected. Nevertheless, the relevant nerves and both arteries could be saved, so that normal forearm function was maintained. The resected tumour measured 140 × 10 × 60 mm, with 110 × 30 mm covering skin tissue. The scar of the previous biopsy was resected. The pathological examination confirmed that the tumour was completely resected and the surrounding tissue was free of malignancy. After dissection by the pathologist, grossly, myxoid and jelly-like shiny regions were seen. Microscopically, necroses were seen in the following staining areas of the tumour: haematoxylin-eosin, periodic-acid Schiff and Elastica van Gieson. Other regions showed cellular areas with pleomorphic oval and spindle shaped cells with hyperchromatic nuclei with many atypical mitosis and apoptosis, as in the previous biopsy.

The resected axillary lymph node measured 20 × 20 × 25 mm. Macroscopically it showed lobulated and necrotic brown tissue. Microscopic findings proved it to be a metastasis of the above-mentioned tumour.

Pathology examination of the tumour in the forearm and the enlarged axillary lymph node confirmed the previous diagnosis of a dedifferentiated high-grade (G3) pT2a liposarcoma with left axillary lymph-node metastases. This correlated with stage IV disease, on the staging system of the American Joint Committee on Cancer.

**Figure 3:**
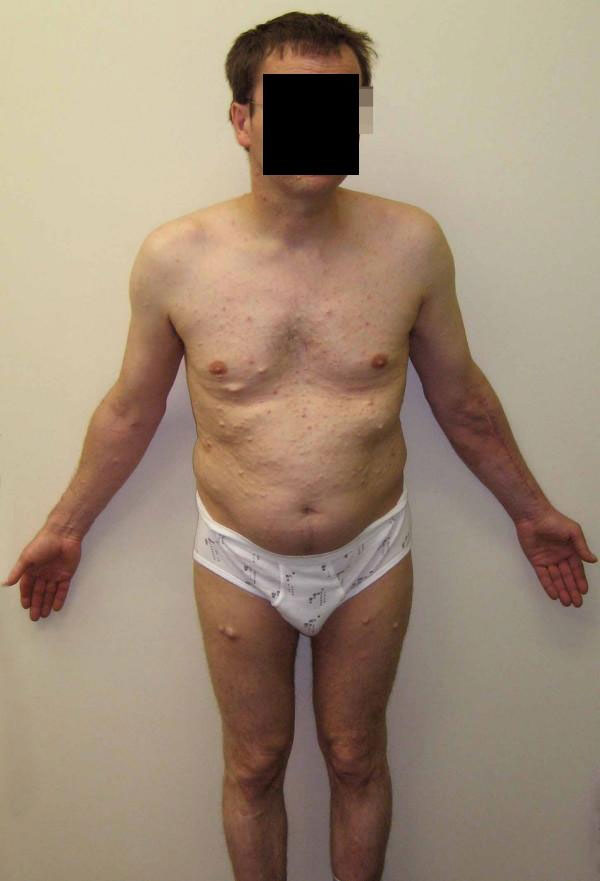
**Generalized neurofibromatosis**. Note the scar on the left forearm after resection of the liposarcoma.

The wounds healed primarly, with no motor, sensory or vascular complications (Figure 3). After successful surgery followed by a 2-week stay in hospital, the patient was discharged. The movement in the operated forearm was free, without limitation.

Ambulant, adjuvant radiotherapy commenced 6 weeks later. The total dose of radiotherapy was determined by normal tissue tolerance. External-beam radiotherapy (1.8 Gy/day, 5 days/week) was given to the left forearm region and to the supraclavicular region with a total dosage of 59.4 Gy. To protect the brachial plexus, the total dosage for the left axillary region was reduced to 50.4 Gy. After extensive discussion with the patient concerning adjuvant chemotherapy, he decided against it for fear of side effects. He has been followed-up and screened continuously every 3 months for possible further malignant transformations or local recurrence.

## Discussion

Liposarcomas are classified into five subtypes according to the World Health Organization: well-differentiated, dedifferentiated, myxoid, pleomorphic and mixed type. Dedifferentiated liposarcomas account for 10% of cases; they occur in the retroperitoneum three times more often than in the extremities [9]. Neoplasms, in association with neurofibromatosis, represent the most serious complication of this disorder [1]-[3]. Malignant transformation in somatic soft tissue as a complication of neurofibromatosis has been studied by D'Agostino et al. [8]. They divided these sarcomas into two types: those clearly arising from a nerve trunk, showing the uniform histology of malignant schwannoma and those arising in somatic soft tissue with or without a demonstrable relationship to a nerve trunk, showing a pleomorphic histology. D'Agostino et al. attribute the pleomorphism to the fact that the neurilemmal cell is multipotential and may undergo metaplasia to fat, bone, cartilage, striated muscle and even osteoid [8]. Hence the occurrence of liopsarcoma in neurofibromatosis patients is possible.

Liposarcoma complicating NF1 is a rare occurrence; until now, only five cases have been reported in the literature [4]-[8]. Our case is a new one to be added to this short list. The most common sites in the body for primary liposarcoma are the lower limb and the retroperitoneum [10]. The localization of known cases of liposarcomas in combination with neurofibromatosis were the lower limb, the omentum and the skull. Our case was located in the upper limb.

Most commonly, a soft-tissue sarcoma presents itself as an asymptomatic mass. The differential diagnosis of a soft-tissue mass includes malignant lesions, desmoids and benign lesions. Our case indicates the importance of considering soft-tissue swelling and growths as malignant until the reverse is proven.

In general, limb-sparing surgery is preferred to achieve local tumour control with minimal morbidity. Both the surgeon and the pathologist should thoroughly document surgical margins, by evaluating a resected specimen.

The recommended treatment for liposarcoma is a radical local excision of the tumour, while trying to preserve the limb. It should be followed by radiotherapy to enhance local control. The effectiveness of adjuvant external beam radiotherapy has been shown in several retrospective and three prospective randomized trials that compared surgery alone to surgery in combination with radiation [10].

Adjuvant chemotherapy treatment of localized dedifferentiated liposarcomas of extremities is controversial because no sufficient and convincing data are available. However, soft-tissue sarcomas are a heterogeneous group. Most published studies include soft-tissue sarcomas without discrimination of the histological type, localization or tumour size. In an Italian randomized cooperative trial, patients with high-grade or recurrent extremity sarcoma showed a better overall survival rate with adjuvant chemotherapy [11]. The recently published review by Dalal et al. is in agreement and suggests that chemotherapy for soft-tissue sarcoma should be regarded as suitable for initial investigation or clinical trials and is rarely indicated, except in carefully selected high-risk patients with high-grade-extremity liposarcoma [10].

Amputation for sarcomas of extremities should only be considered in cases of extensive soft-tissue mass, skin involvement or recurrence prior to adjuvant radiation [12].

In our case, there were axillary metastases, but the decision not to amputate was taken intra-operatively after assuring that an R_0_ resection was possible. The treatment was completed with postoperative radiotherapy. Due to the large tumour size, histopathological findings and lymph-node metastases, we recommended adjuvant chemotherapy, which the patient decided against.

Concerning the treatment of soft tissue sarcoma, local control rates of 85% to 90% have been achieved with a combination therapy of surgery and radiation [13]. Discussion is ongoing concerning the timing of radiation; that is, whether it should be given before or after surgery [13]. Pre-operative radiation has the advantage that the tumour may shrink in size, making the surgery technically more feasible. The downside is an increased possibility of wound complications. Pollack et al. reported wound-healing complications in 25% in patients who received radiotherapy pre-operatively, versus 6% in those with postoperative treatment [14]. The role of chemotherapy in the treatment of liposarcoma remains controversial and is best addressed on a case-by-case basis [13,15].

## Conclusion

Despite its rarity, we present this case to stress the importance of liposarcoma as a potential complication in neurofibromatosis. This emphasizes the relevance of an early detection of malignancy in increased swelling in patients with NF1. Keeping this in mind could save patients from major surgery and tumour complications.

Concerning the treatment, we recommend early intensive investigations and an interdisciplinary approach, such as a tumour board, regarding surgery, radiotherapy, chemotherapy and further treatment. The patient should present for follow-up regularly.

## Abbreviations

NF1: neurofibromatosis type 1; MRI: magnetic resonance imaging.

## Consent

Written informed consent was obtained from the patient for publication of this case report and accompanying images. A copy of the written consent is available for review by the Editor-in-chief of this journal.

## Competing interests

The authors declare that they have no competing interests.

## Authors' contributions

MDS, MYAS, SFW, JP and BFEZ all analyzed and interpreted the patient data regarding the NF1 and liposarcoma. MDS carried out the operation on the patient and was the main contributor in the writing of the manuscript. All authors read and approved the final manuscript.
